# Peliosis Hepatis Simulates Liver Metastases

**DOI:** 10.1200/JGO.2016.008839

**Published:** 2017-02-15

**Authors:** Bruna Migliavacca Zucchetti, Andrea Shimada, Luiz Tenorio Siqueira

**Affiliations:** **Bruna Migliavacca Zucchetti** and **Andrea Shimada,** Hospital Sirio-Libanês; and **Luiz** **Tenorio Siqueira,** Hospital Nossa Senhora das Graças, São Paulo, Brazil.

## CLINICAL CASE

A 68-year-old Japanese woman was referred for evaluation to our service in September 2013; she complained of abnormal uterine bleeding. She was 10 years postmenopausal, nulliparous, and otherwise healthy except for a corneal transplantation in her right eye in 2002.

She underwent a few investigational examinations, and her transvaginal ultrasound showed an abnormally thickened endometrium. In the hysteroscopy, she had an enlarged uterus with an irregular endometrial lining and a few uterine polyps that bled easily. The endometrial biopsy was consistent with an endometrial adenocarcinoma, histologic grade 1, nuclear grade 2, and neoplastic myometrium infiltration.

Her pelvic magnetic resonance imaging (MRI) results showed uterine myomas and a thick and heterogenic endometrium that measured 1.2 cm. The chest computed tomography (CT) scan had multiple bilateral nodules that were randomly distributed in both lungs, which was suggestive of metastatic disease. A CT-guided biopsy of one of the pulmonary nodules was performed, and the histologic result was metastatic endometrial adenocarcinoma. The immunohistochemistry was β-catenin negative, thyroid transcription factor 1 negative, progesterone receptor and estrogen receptor positive, vimentin negative, CK7 positive, and carcinoembryonic antigen negative.

To control the uterine bleeding, megestrol acetate 160 mg daily was prescribed in December 2013; however, the bleeding did not stop completely, so the patient underwent a total hysterectomy for local control in February 2014. The pathologic analysis of the uterus confirmed an endometrial adenocarcinoma, moderately differentiated, histologic grade 1, with infiltrations of more than two-thirds the depth of the myometrium and with vascular invasion. The final pathologic staging was pT1bNxM1.

**Fig 1:**
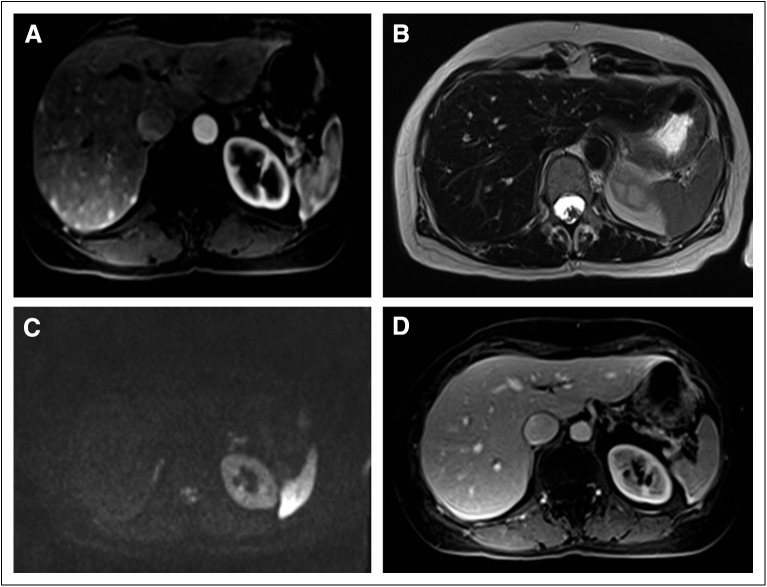
Abdominal magnetic resonance imaging. (A) Three-dimensional gradient echo (GRE) T1 volumetric interpolated breath-hold examination (VIBE) sequence with gadolinium demonstrates multiple arterial-enhancing foci scattered throughout the right lobe of the liver. (B) Fast-spin echo T2-weighted sequence without fat suppression shows normal hepatic parenchyma with no evidence of focal lesions. (C) Diffusion-weighted images with no focal lesions within the liver. (D) Three-dimensional GRE T1 VIBE sequence portal phase also demonstrates no evidence of liver lesions.

Also in February 2014, the patient underwent an abdominal and pelvic MRI (Fig 1) that indicated the presence of highly vascularized liver nodules that were localized mostly in the right lobe, had lack of perfusion in the adjacent parenchyma, and measured approximately 0.8 cm. These lesions did not appear to be metastatic nodules, and vascular abnormalities were considered.

At this time, her physical exam and the results of routine laboratory investigations, including liver enzymes, were unremarkable, so we decided to proceed with a conservative approach of regular follow-up visits. The patient continued to take megestrol acetate 160 mg daily and remained asymptomatic.

An abdominal MRI was repeated every 3 months, and the liver nodules did not change in number or size until March 2015, when the MRI indicated growth of the nodules from 0.8 cm to 1.4 cm. At this time, a percutaneous needle biopsy of one of the liver nodules was done. The histologic result showed a cystic lesion filled with erythrocytes throughout the lobule, with moderated sinusoidal dilatation and atrophy of the adjacent hepatocytes. The portal space had a few lymphocyte infiltrations, and there was no fibrosis or any malignancy in this sample. The final pathologic diagnosis was peliosis hepatis. Megestrol acetate is associated with peliosis hepatis, so the patient was prescribed anastrozole 1 mg daily instead in April 2015.

The patient remains asymptomatic and undergoes a repeat abdominal MRI every 3 months. The last evaluation was in July 2016, and the hepatic nodules were stable.

## DISCUSSION

Peliosis hepatis was first reported in the German literature in 1861 by Wagner and was named by Schoelank in 1916.^1^

The pathogenesis of peliosis hepatis is still unclear. It involves hepatocellular necrosis and injury in the sinusoidal endothelium.^1^ One of the hypotheses is that the necrosis destroys the reticulum framework, which causes hemorrhage; it can heal and form a cyst.^1^

Some clinical conditions, such as HIV infection, *Bartonella henselae* or *Bartonella quintana* infection, syphilis, and tuberculosis, are associated with the development of peliosis hepatis.^2^ Some drugs, such as mercaptopurine, androgenic-anabolic steroids, danazol, glucocorticoids, tamoxifen, and oral contraceptives,^1^ can cause peliosis hepatis.

In our research, we found some relation between peliosis hepatis and malignancies, especially the hematologic ones. To the best of our knowledge, though, this is the first report of a case in which peliosis hepatis was diagnosed in a patient with endometrial adenocarcinoma who had been treated with a progesterone analog.

The natural history of peliosis hepatis is poorly understood. The clinical presentation and laboratory data are nonspecific and depend on the disease process.^2^ Most patients are asymptomatic or have a slowly progressive disease, but there are reports of portal hypertension and of spontaneous bleeding that causes intrahepatic and peritoneal hemorrhage.^1^

Currently, by using modern cross-sectional imaging studies, the diagnosis of peliosis hepatis is increasing, especially in asymptomatic patients. The imaging appearance of peliosis hepatis is difficult to differentiate from multiple abscesses in the liver, adenoma, focal nodular hyperplasia, hemangiomatosis, or liver metastases.^3^

At CT scan, the peliosis hepatis enhancement pattern varies, depending on the freshness of the blood that fills the peliotic cavities. Fresh blood is associated with marked enhancement, whereas retention of old blood is associated with little or no enhancement. The MRI findings include T1 hypointense and T2 hyperintense lesions, which show early peripheral and late diffuse contrast enhancement on dynamics imaging.^4^

The definitive diagnosis of peliosis hepatis is established by histopathology. Therefore, a percutaneous needle biopsy can confirm the diagnosis.^5^ However, even with ultrasound or CT-guided biopsy, the procedure has a high risk of life-threatening hemorrhage.^5^ Microscopic exam of peliosis hepatis reveals round or oval intralobular cavities that are randomly distributed between areas of normal hepatic parenchyma. The cavities communicate with sinusoids that are sometimes dilated.^1^ Red blood cells can be seen in the peliotic cysts.

There is no specific treatment of peliosis hepatis except for antibiotics used to treat occurrences related to *Bartonella* infections. For diagnoses of peliosis hepatis without an infectious cause, early detection and discontinuation of the causative agent or treatment of the condition that causes the peliosis hepatis may result in regression of the hepatic lesions. Rare occurrences with liver failure require liver transplantation.^1^

In our research, we could not find guidelines to monitor patients with peliosis hepatis. Liver biochemical exams and repeated liver imaging studies can be useful to evaluate disease progression or regression.

In conclusion, clinicians and radiologists must recognize these lesions to minimize the probability of misdiagnosis and inappropriate treatment. It is likely that peliosis hepatis is underdiagnosed in radiologic studies. Peliosis hepatis should be considered in the differential diagnosis of liver lesions in patients with cancer when the clinical setting does not seem to indicate metastases.
